# Overexpression of glycolysis markers in placental tissue of pregnant women with chronic venous disease: a histological study

**DOI:** 10.7150/ijms.65419

**Published:** 2022-01-01

**Authors:** Miguel A Ortega, Miguel A Sáez, Oscar Fraile-Martínez, Miguel A Álvarez-Mon, Cielo García-Montero, Luis G Guijarro, Ángel Asúnsolo, Melchor Álvarez-Mon, Julia Bujan, Natalio García-Honduvilla, Juan A De León-Luis, Coral Bravo

**Affiliations:** 1 Department of Medicine and Medical Specialties, Faculty of Medicine and Health Sciences, University of Alcalá, Alcalá de Henares, Madrid, Spain; 2 Ramón y Cajal Institute of Healthcare Research (IRYCIS), Madrid, Spain; 3 Cancer Registry and Pathology Department, Hospital Universitario Principe de Asturias, Alcalá de Henares, Spain.; 4 Pathological Anatomy Service, Central University Hospital of Defence-UAH Madrid, Spain; 5 Service of Gynecology and Obstetrics, Section of Fetal Maternal Medicine, Central University Hospital of Defence-UAH Madrid, Spain; 6 Unit of Biochemistry and Molecular Biology (CIBEREHD), Department of System Biology, University of Alcalá, 28801 Alcalá de Henares, Spain; 7 Department of Surgery, Medical and Social Sciences, Faculty of Medicine and Health Sciences, University of Alcalá, Alcalá de Henares, Madrid, Spain; 8 Immune System Diseases-Rheumatology and Oncology Service, University Hospital Príncipe de Asturias, CIBEREHD, Alcalá de Henares, Madrid, Spain; 9 Department of Public and Maternal and Child Health, School of Medicine, Complutense University of Madrid, 28040 Madrid, Spain; 10 Department of Obstetrics and Gynecology, University Hospital Gregorio Marañón, Madrid 28009, Spain; 11 Health Research Institute Gregorio Marañón, 28009 Madrid, Spain; + These authors contributed equally to this work.

**Keywords:** Chronic venous disease (CVD), Placenta, Glucose Homeostasis, Metabolomic profiling, Glycolytic phenotype

## Abstract

Chronic Venous Disease (CVD) refers to a wide variety of venous disorders being the varicose veins its most common manifestation. It is well-established the link between pregnancy and the risk of suffering CVD, due to hormonal or haematological factors, especially during the third trimester. In the same manner, previous studies have demonstrated the detrimental effect of this condition in the placental tissue of pregnant women, including in the normal physiology and the metabolomic profile of this organ. In this context, the aim of this study was to evaluate the glucose homeostasis in the placental tissue of women presenting CVD. Through immunohistochemistry, we studied the protein expression of the glucose transporter 1 (GLUT-1), Phosphoglycerate kinase 1 (PGK1), aldolase (ALD), Glyceraldehyde-3-phosphate dehydrogenase (GA3PDH) and lactate dehydrogenase (LDH). Our results have reported a significative increase in the expression of GLUT-1, PGK1, ALD, GA3PDH and the isoenzyme LDHA in placentas of women with CVD. This work has proven for the first-time an altered glucose metabolism in the placental tissue of women affected by CVD, what may aid to understand the pathophysiological mechanisms of this condition in more distant organs such as placenta. Furthermore, our research also supports the basis for further studies in the metabolic phenotyping of the human placenta due to CVD, which may be considered during the late pregnancy in these women.

## Introduction

Chronic venous disease (CVD) defines a broad spectrum of venous disorders from mild to severe clinical manifestations which encompass a remarkable inflammatory response secondary to a situation of venous hypertension 1. Varicose veins (VVs) are the most frequent presentation of CVD, generally affecting elder people and women 2. Although it seems that genetics may play a significant role in the establishment of CVD 3, other factors such as body mass index (BMI) > 25 kg/m2 smoking, menopause or physical inactivity are also related with an increase in the occurrence of CVD 4,5. Pregnancy is a period associated with many changes in the women´s body including at cardiovascular, renal, respiratory, endocrine and metabolic levels 6. Despite these modifications are needed for the successful development of the foetus and gestation, pregnancy is equally presumed to be a major contributor of CVD 7. Some of the proposed mechanisms are haemodynamic alterations and hormonal interactions during pregnancy, to the structure and adequate functioning of the vein, particularly in the lower limbs 8-10.

Previous research indicates that, not only pregnancy may promote the onset of CVD, but also this condition might interact with the proper development of pregnancy. Particularly, it has been studied the effect of CVD in the placenta of women affecting by these disorders, finding increased calcifications in the placental villi 11, altered angiogenesis and lymphangiogenesis 12 or an augmentation in the oxidative stress markers 13, therefore denoting the detrimental impact of CVD during pregnancy.

Recent studies have shown the imperial need of addressing the metabolic phenotyping in venous pathology 14. Noteworthy changes in the placental metabolic profile have been reported in preeclampsia, a condition characterised by a pathological arterial hypertension 15. Similarly, we have just conducted a research demonstrating an abnormal lipidomic profile in placentas of women with CVD 16, hence assuming the effect of this condition in the metabolic phenotyping of the tissue. In this context, glucose metabolism appears to have a central role in the normal physiology of the placenta, particularly during the first stages of pregnancy 17. Although at physiological conditions placenta perform an oxidative metabolism, it is an easily adapted organ capable of modulating its metabolic phenotype under stressful conditions to assure the success of the pregnancy 18. For instance, an altered glycolysis and mitochondrial oxidative phosphorylation have been postulated to take part in the pathophysiology of intrauterine growth restriction 19. In this line, we hypothesize that the pathological environment caused by CVD in the placenta may affect the metabolic status of this organ. Thus, the aim of this study is to analyse the glycolytic phenotype of placentas of women with CVD regarding its intake and metabolism. With that purpose we examined the role of Glucose Transporter 1 (GLUT-1), and the glycolytic enzymes Phosphoglycerate kinase 1 (PGK1), aldolase (ALD), Glyceraldehyde-3-phosphate dehydrogenase (GA3PDH) and the lactate-producer enzyme lactate dehydrogenase (LDH) studying their protein expression through the development of immunohistochemistry.

## Patients and Methods

### Study population

An observational, analytical and prospective study was conducted, where 114 pregnant women were evaluated in the third trimester of pregnancy (32 weeks). 62 were diagnosed with CVD, with an average age of 33 22-40 and a median gestational time of 40.5 [39-41.5] weeks for all clinical and histological studies. Likewise, 52 control women with no presence of CVD (HC) were studied, with a median age of 34 27-41 years and a median gestational time of 41 39-42 weeks for all clinical and histological studies. This study has been conducted following the four basic ethical principles: Autonomy, beneficence, non-maleficence and distributive justice. This work was developed according to the standards of Good Clinical Research Practice, the principles set forth in the last Declaration of Helsinki (2013), and the Oviedo Convention (1997). Patients were properly informed, each one providing their signed consent. The project was approved by the the Clinical Research Ethics Committee of the Gómez-Ulla-UAH Defence Hospital (37/17). During the third trimester visit clinical history is collected, along with a general physical exam and a lower extremity exploration with an Eco-Doppler (Portable M-Turbo Eco-Doppler; SonoSite, Inc., Washington, EEUU) at 7,5 MHz. Inclusion criteria were women over 18 years during the third trimester of pregnancy with clinical evidence of lower limb CVD were included, with CEAP (Clinical-Etiology-Anatomy-Pathophysiology) ≥1 20. Exclusion criteria were women diagnosed with diabetes mellitus or other metabolic disorders, arterial hypertension (AHT), autoimmune diseases, active infections, venous malformations, cardiac, renal or lung insufficiency, preeclampsia or Hellp syndrome, intrauterine growth restriction by known causes body mass index (BMI) ≥ 25 kg/m^2^, presence of pathological lesions like placental infarcts, delayed maturation, avascular villi, and chronic inflammation affecting chorionic villi, together with the appearance of any of the previous exclusion criteria any time before to delivery; as well as prior evidence of CVD.

### Tissue samples

Placental biopsies were obtained after delivery. In all cases, placenta was cut in 5 fragments by using a scalpel to assure that the sample includes various cotyledons (placentons). Afterwards, these fragments were introduced in a sterile tube containing Minimum Essential Medium (MEM) with a 1% antibiotic/antimycotic (both from ThermoFisher Scientific, Waltham, MA, EEUU). All samples were transferred viarefrigeration to the laboratorywithin 2 hours after delivery. In the laboratory, the samples were processed in a laminar class II laminar flow hood (Telstar AV 30/70 Müller 220 V 50 MHz; Telstar SA Group, Terrassa, Spain), in a sterile environment. Then, MEM samples were destined to immunodetection and histological studies.

Placental fragments preserved in MEM were washed and hydrated multiple times, with antibiotic-free medium, removing blood cells, then divided into fragments, and subsequently fixed in F13 (60% ethanol, 20% methanol, 7% polyethylene glycol, 13% distilled H2O), following established protocols 12. Once included, paraffin blocks were made using molds. After paraffin's solidification, an HM 350 S rotation microtome (Thermo Fisher Scientific, MA, USA) was used to produce 5 µm thick sections, taking it to a hot water bath, to be collected on a glass slide, previously treated with 10% polylysine for improving adhesion of the cuts.

### Immunohistochemistry studies

Detection of antigen-antibody reaction was studied by the method ABC (Avidin-biotin complex) with peroxidase as chromogen in agreement with the protocol of Ortega et al. 21. The incubation with primary antibody was diluted in 3% BSA and PBS during the whole night at 4ºC (Table 1A). On the other hand, incubation with the secondary antibody bound to biotin and diluted in PBS was conducted during an hour and a half at room temperature (Table 1B). Then, ExtrAvidin®-Peroxidase (Sigma-Aldrich, St. Louis, MO, EEUU) conjugated was used for 60 minutes at room temperature (in a PBS 1:200 dilution), revealed with the chromogenic substrate diaminobenzidine (Kit DAB, SK-4100, Vector Laboratories, Burlingame, CA, EEUU), prepared immediately before exposure (5 mL of distilled water, two drops of buffer, four drops of DAB, two drops of hydrogen peroxide). This procedure allows a brown staining. In every immunohistochemistry, sections from the same tissue were used as negative controls, in which the incubation with primary antibody was substituted with incubation in a blocking solution (PBS).

### Statistical analysis and microscopic examination

For the statistical analysis, GraphPad Prism® 6.0 was used to conduct a Whitney U test. Data are expressed as the median with interquartile range (IQR). The significance was established at a value of p <0,05 (*), p <0,01 (**), p <0,001 (***). To quantify the immunopositive cells, 5 counts were randomly applied to tissue sections, excluding cells not entering the designated demarcation lines. The percentage of positive cells was calculated by the following formula: % Positive cells = No. Cells+/(No. Cells- +No. Cells-) × 100. Patients were described as positive when theme an of the test sample scored for each subject was greater than or equal to 5% over the total , following the anatomo-pathological criteria stated by Cristóbal et al. 22 this procedure is a minimal modification of the immunoreactive score (ISR score). Immunostaining in the tissue was assessed by two independent histologists (M.AO. and M.A.S.) blinded to the outcome measure. The cuts were studied under a Zeiss Axiophot optical microscope (Carl Zeiss, Germany).

## Results

**Figure 1:**
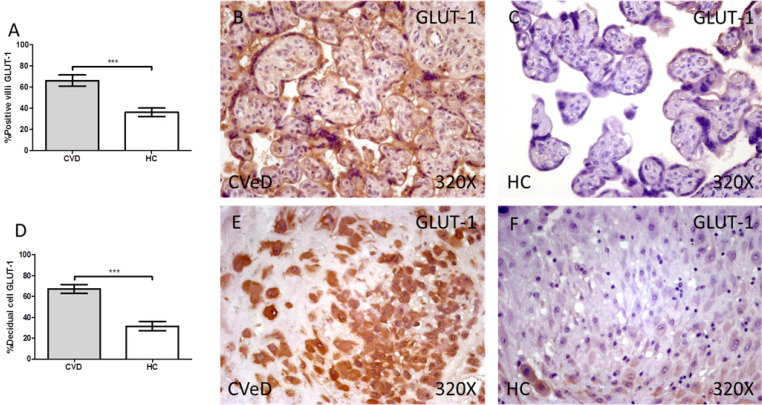
Percentage of placental villa (A) and decidual cells (D) with positive expression for GLUT-1 through the use of immunohistochemistry techniques. B-F. Images showing immunoexpression of GLUT-1 in the placental villi (B and C) and decidual cells (E and F). CVD=Women diagnosed with gestational chronic venous disease. HC= Control without venous pathology. n=62 with CVD and n=52 control women with no presence of CVD (HC) were studied for all clinical and histological studies. p<0.005 (*),p <0,01 (**), p<0.001(***)

We have reported a significative increase through immunohistochemistry techniques in the protein expression of GLUT-1 in the placenta of women with CVD when comparing with HC. This expression was prominently superior in placental villi of CVD (CVD = 65.00 [12.00-99.00] vs HC = 36.00 [9.00-74.00], ***p < 0.0001, Figure 1A-C). In the same line, we have observed a significative augmentation in the expression of GLUT-1 in the decidual cells of CVD placentas (CVeD = 67.00 [21.00-99.00] vs HC = 27.00 [10.00-74.00], ***p < 0.0001, Figure 1D-F).

**Figure 2:**
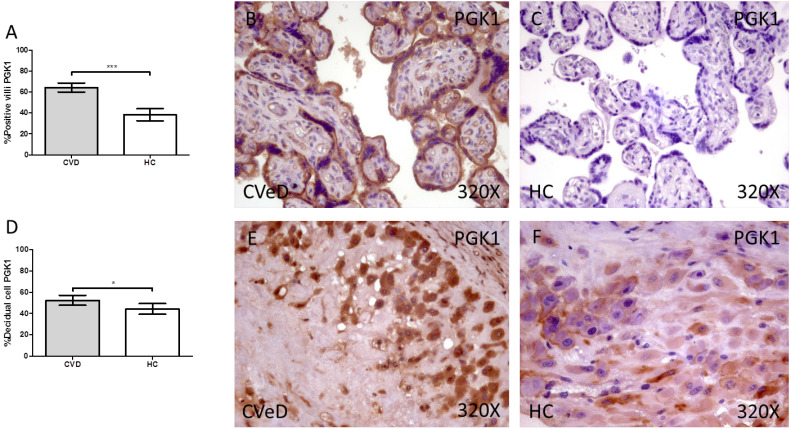
Percentage of placental villa (A) and decidual cells (D) with positive expression for PGK1 through the use of immunohistochemistry techniques. B-F. Images showing immunoexpression of PGK1 in the placental villi (B and C) and decidual cells (E and F). CVD=Women diagnosed with gestational chronic venous disease. HC= Control without venous pathology. n=62 with CVD and n=52 control women with no presence of CVD (HC) were studied for all clinical and histological studies. p<0.005 (*),p <0,01 (**), p<0.001(***)

Placentas of women with gestational CVD showed a significative increase in the protein expression of PGK1. In this sense, we have detected higher levels of placental villi with positive protein expression for PGK1 in comparison to HC (CVD = 64.00 [25.00-97.00] vs HC = 34.00 [3.00-85.00], ***p < 0.0001, Figure 2A-C). Furthermore, the percentage of decidual cells expressing this component is significatively superior in women with CVD than HC (CVD = 52.00 [12.00 - 91.00] vs HC = 46.00 [7.00-74.00], *p = 0.0460, Figure 2D-F).

**Figure 3:**
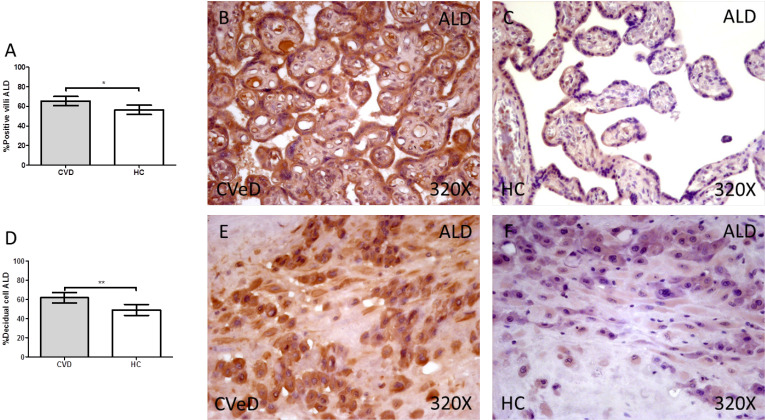
Percentage of placental villa (A) and decidual cells (D) with positive expression for ALD through the use of immunohistochemistry techniques. B-F. Images showing immunoexpression of ALD in the placental villi (B and C) and decidual cells (E and F). CVD=Women diagnosed with gestational chronic venous disease. HC= Control without venous pathology. n=62 with CVD and n=52 control women with no presence of CVD (HC) were studied for all clinical and histological studies. p<0.005 (*),p <0,01 (**), p<0.001(***)

Lastly, our results indicate a significative augmentation of the enzyme ALD in placentas of women with gestational CVD (CVD=63.50 [25.00-98.00] vs HC = 56.00 [20.00-91.00], *p = 0.0115, Figure 3.A-C). The abovementioned expression was significatively higher in the placental villi of women with CVD as well as in the decidual cells in comparison to HC (CVD = 64.00 [18.00-96.00] vs HC = 46.00 [11.00-89.00], *p = 0.0013, Figure 3D-F).

**Figure 4:**
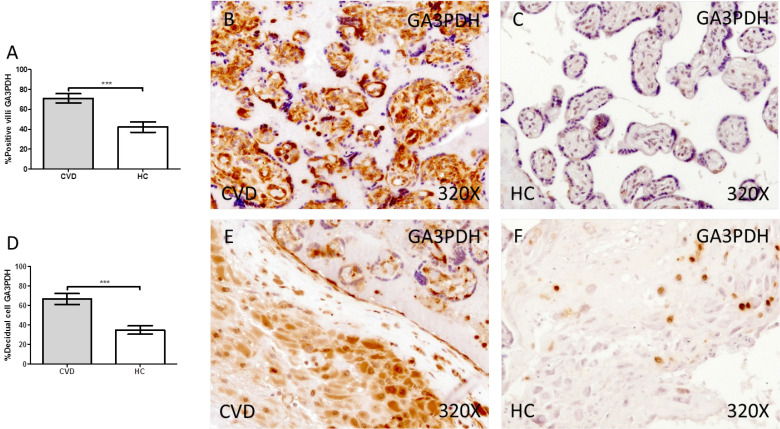
Percentage of placental villa (A) and decidual cells (D) with positive expression for GA3PDH through the use of immunohistochemistry techniques. B-F. Images showing immunoexpression of GA3PDH in the placental villi (B and C) and decidual cells (E and F). CVD=Women diagnosed with gestational chronic venous disease. HC= Control without venous pathology. n=62 with CVD and n=52 control women with no presence of CVD (HC) were studied for all clinical and histological studies. p<0.005 (*),p <0,01 (**), p<0.001(***)

Our results have shown a significant increase in the protein expression of GA3PDH in the placental villi of women with gestational CVD compared to HC (CVD = 74.00 [24.00-98.00] vs HC = 41.50 [10.00-85.00], *** p < 0.0001, Figure 4.A-C). Similarly, our results have shown a significant increase in GA3PDH in the decidual cells of the placentas of women with gestational CVD compared to HC (CVD = 65.00 [21.00-99.00] vs HC = 34.50 [10.00-74.00], ** * p < 0.0001, Figure 4.D-F).

**Figure 5:**
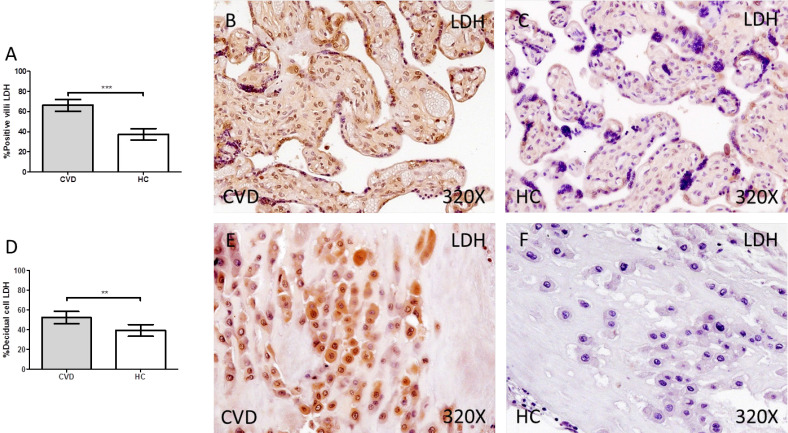
Percentage of placental villa (A) and decidual cells (D) with positive expression for LDH through the use of immunohistochemistry techniques. B-F. Images showing immunoexpression of LDH in the placental villi (B and C) and decidual cells (E and F). CVD=Women diagnosed with gestational chronic venous disease. HC= Control without venous pathology. n=62 with CVD and n=52 control women with no presence of CVD (HC) were studied for all clinical and histological studies. p<0.005 (*),p <0,01 (**), p<0.001(***)

Finally, we have observed a significant increase in the protein expression of LDH in the placental villi of women with gestational CVD compared to HC (CVD = 74.00 [10.00-99.00] vs HC = 33.00 [12.00-85.00], *** p < 0.0001, Figure 5A-C). In parallel, we observed a significant increase in LDH in the decidual cells of the placentas of women with gestational CVD compared to HC (CVD = 54.50 [12.00-98.00] vs HC = 34.50 [10.00-94.00], ** p = 0.0083, Figure 5D-F).

## Discussion

CVD is a condition characterised by venous hypertension that could affect to the normal environment of placenta and its metabolic profile 16,23. For the first time we have detected an altered glycolytic phenotype in placentas of women with CVD, demonstrated by an increase in the expression of GLUT-1, PGK1, ALD, GA3PDH and LDHA, five critical components involved in the transport and metabolism of glucose.

Different factors like nutritional state, maternal stress or oxygen availability appears to play a central role in the adaptations of human placenta, hence determining its metabolic phenotyping 24. In this sense, we previously reported an increased hypoxia in the placental villi of women presenting CVD 25, which may help to explain the alterations observed in our study. It has been described the metabolic reprogramming of placenta under conditions of hypoxia, including in the expression of glycolytic genes 26. This hypoxia may interact with other factors altered in placentas of women with CVD such as the Vascular Endothelial Growth Factor (VEGF) and Pigment Epithelium-derived Factor (PEDF) therefore promoting angiogenesis and glucose metabolism 11,27. Interestingly, this behaviour reported in the placenta is similar to the mechanisms occurring in the tumoral cells in a process known as Warburg effect, leading to an increased proliferation, reduced cell death, vascular remodeling, invasiveness and immune system communication 28. Nonetheless, in the placental tissue hypoxia seems to be important during the first stages of pregnancy, being progressively decreasing during the gestation, contrary to tumours, where it sustainedly increases 29. Our results are consistent with those obtained by Wei & Hu 30, who detected a marked augmentation in some glycolytic genes including GLUT-1 and PGK1 in placentas of women with intrahepatic cholestasis due to a marked hypoxia. On the other hand, the possible consequences of the low oxygen environment and its glycolytic response remain to be elucidated and it will depend on the timing of exposure, the severity and the duration of hypoxia 31. Howsoever, our study suggests an important alteration in the normal behaviour of placenta in women with CVD probably determined by previous reported hypoxia.

Therefore, glycolytic phenotyping determines a proliferative status in the placenta of women with CVD. This status is similar to those occurring in some adverse outcomes of pregnancy such as maternal diabetes 32. This could be due to the pathological environment of placenta in women with CVD which leads to a noteworthy elevation of apoptosis due to this condition 25 and glycolytic switch may be a response of placenta to supply this altered process. Oxidative stress, also detected in women with CVD 13 may promote a synergic response with hypoxia in the glycolytic phenotyping of placenta 33. GLUT- 1 is the main member of the glucose transporters in the placenta, highly expressed during the various stages of pregnancy 34. Although it is tightly regulated by extracellular glucose levels, altered levels of GLUT-1 has been observed in some pregnancy complications 35,36 thereby assuming the relevant role of this component in the placenta. It has been demonstrated the important action of Insulin-like growth factor I (IGF-I) in the increased levels of GLUT-1, stimulating basal membrane transport of glucose, leading to augmented transepithelial glucose transport 37. Also, PI3K/Akt pathway may take part in the glycolytic switch suffered by the placenta of women with CVD 38. This route is hyperactivated during hypoxia and promotes the activity of different components involved in the metabolic reprogramming of proliferative cells, for instance through the activation of the enzyme ALD 39 or the proper PGK1 40, negatively regulated by the natural inhibitor of PI3K pathway, PTEN. The importance of these components was found in the venous walls of VVs 41,42, so it is likely that the alteration of these components might be implicated in the changes occurred in the placenta of women with CVD. Furthermore, we have observed a significant increase of GA3PDH and the isoform LDHA in the placenta of women with CVD. GA3PDH is responsible for the formation of 2 molecules of NADH+H^+^ in glycolysis. These 2 NADH+H^+^ may be used either for aerobic respiration, accounting for 6 ATPs or it could mediate the transformation from pyruvate to lactate by the LDH 43. Altogether, the significant upregulation of the glucose transporter GLUT-1, and the glycolytic enzymes ALD, PGK1, GA3PDH and the isoform LDHA appears to define a glycolytic switch in the placenta of women with CVD. Moreover, the augmented detection of GA3PDH indicates that a high production of NADPH+H^+^ exists in this which is mainly directed to lactate formation, as showed by an increase in the isoenzyme LDHA. This increased expression of the isoenzyme LDHA and lactate production has also been observed in the pathogenesis of other placental vascular pathologies such as pre-eclampsia 44. Interestingly, there are multiple studies evidencing the promising use of serum LDH as a diagnostic and prognostic factor in this obstetrical condition 45,46. Future studies may be directed to analyze serum levels of this enzyme in women with CVD and its possible clinical correlations.

Finally, it is hard to establish if this glycolytic is a cause or a consequence of the placental damage. Probably, both are right. For instance, lactate is a pivotal molecule in early stages of pregnancy, where it may exert immunomodulatory actions, also participating in other processes like angiogenesis 47. The increased lactate production may result from an adaptative response of the placenta to the abnormal environment previously observed in this tissue. In turn, changes in the glucose metabolism may conduct to substantial alterations in the cell cycle 48 and lipid metabolism 49, both observed in the placenta of patients with CVD 16,50. Further efforts should be placed to unravel possible translational and clinical implications of the glycolytic switch in placentas of women with CVD.

## Conclusions

Overall, our study is the first to demonstrate the altered metabolic status in the placental tissue of women with CVD which conducts to an increased glycolysis reported by the higher detection of GLUT-1, PGK1, ALD, GA3PDH and LDH. This could be considered as a consequence of the pathological environment found in the placenta of women with CVD, thereby interfering with the normal metabolism of this organ. Further studies will be needed to unravel the possible mechanisms related with these changes as well as the possible consequences of the metabolic reprogramming of these placentas according to maternal and foetal outcomes.

## Figures and Tables

**Table 1A:** The different antibodies (A. primary, B. secondary) used in the immunohistochemistry studies performed with dilutions and protocol features.

Antigen	Species	Dilution	Provider	Protocol specifications
**PGK1**	Rabbit	1:500	Abcam (ab154613)	10 mM Sodium citrate pH = 6 before the incubation with the blocking solution.
**GLUT1**	Mouse	1: 200	Abcam (ab40084)	10 mM Sodium citrate pH = 6 before the incubation with the blocking solution.
**STC-2**	Rabbit	1:250	Abcam (ab252953)	0,1% Triton with PBS, 10 minutes, before incubation with the blocking solution
**GA3PDH**	Rabbit	1:500	Abcam (ab 134187)	0,1% Triton with PBS, 10 minutes, before incubation with the blocking solution
**LDH**	Rabbit	1:750	Abcam (ab 199553)	0,1% Triton with PBS, 10 minutes, before incubation with the blocking solution

**Table 1B:** The different antibodies (A. primary, B. secondary) used in the immunohistochemistry studies performed with dilutions and protocol features.

Antigen	Species	Dilution	Provider	Protocol specifications
**IgG (Mouse)**	Goat	1:300	Sigma (F2012/045K6072 )	--------------------
**IgG (Rabbit)**	Mouse	1:1000	Sigma (RG-96/ B5283)	--------------------
